# Preparation of Zeolitic Imidazolate Framework-8-Based Nanofiber Composites for Carbon Dioxide Adsorption

**DOI:** 10.3390/nano12091492

**Published:** 2022-04-28

**Authors:** Yu-Chun Chiang, Wei-Ting Chin

**Affiliations:** 1 Department of Mechanical Engineering, Yuan Ze University, Taoyuan 320, Taiwan; s1060806@mail.yzu.edu.tw; 2 Fuel Cell Center, College of Engineering, Yuan Ze University, Taoyuan 320, Taiwan

**Keywords:** zeolitic imidazolate framework, heat treatment, electrospinning, carbon nanofibers, carbon dioxide

## Abstract

In this study, polyacrylonitrile (PAN)-based activated nanofiber composites, which were embedded inside zeolitic imidazolate framework-8 (ZIF-8) crystals or ZIF-8-derived carbons (ZDC-850), were fabricated using an electrospinning process, to serve as CO_2_ adsorbents. The adsorbents were characterized using various techniques. The degree of crystallinity of ZDC-850 totally changed compared to that of ZIF-8. For nanofiber composites, the timing of the ligand decomposition of ZIF-8 significantly affected the material properties. The Zn metals in the ZIF-8/PAN or ZDC-850/PAN could be embedded and protected by the PAN fibers from excess volatilization in the following treatments: ZIF-8 had significant pore volumes in the range of 0.9–1.3 nm, but ZDC-850 and ZIF-8/PAN exhibited a distinct peak at approximately 0.5 nm. The CO_2_ adsorption capacities at 25 °C and 1 atm followed the order: ZIF-8/PAN (4.20 mmol/g) > ZDC-850 (3.50 mmol/g) > ZDC-850/PAN (3.38 mmol/g) > PAN (2.91 mmol/g) > ZIF-8 (0.88 mmol/g). The slope in the log–linear plot of isosteric heat of adsorption was highly associated with CO_2_ adsorption performance. Under 1 atm at 25 °C, for Zn metal active sites inside the pores, the pores at approximately 0.5 nm and in C-N (amines) groups could promote CO_2_ adsorption. At low CO_2_ pressures, for a good CO_2_ adsorbent, the carbon content in the adsorbent should be higher than a threshold value. Under this condition, the percentage of ultra-micropore and micropore volumes, as well as the functional groups, such as the quaternary or protonated N (amines), N=C (imines or pyridine-type N), C-OH, and -COOH groups, should be considered as significant factors for CO_2_ adsorption.

## 1. Introduction

Carbon dioxide emission reduction has become the consensus of many countries. Therefore, the development of effective adsorbents for CO_2_ capture is a top priority. It is known that good CO_2_ adsorbents are dependent on pore volumes of sizes less than 0.7 nm and the presence of surface functional groups [1,2,3,4,5]. Various porous materials have been intensively studied, such as carbon nanofibers, zeolitic imidazolate frameworks (ZIFs), metal organic frameworks, carbon nanotubes, silica, activated carbon fibers, zeolites, and activated carbons.

ZIFs are a subgroup of MOFs, with perpetual porosity consisting of metals bridging to imidazolate-like ligands, with the tetrahedral arrangement as molecular structural blocks [6,7]. The bond angles are 145° [6], similar to the Si–O–Si bond angle in zeolites [8,9,10,11]. These building units are congregated and assemble in large molecular configurations. They have emerged as novel crystalline porous materials. Due to possessing features of both MOFs and zeolites, ZIFs have several unique characteristics, including unimodal micropores, high surface areas, abundant functionalities, high thermal stability, and remarkable chemical robustness. [12]. Therefore, ZIFs have gained a large amount of attention for many potential applications, such as gas separation/adsorption [13,14], water treatment [15], catalysis [16], chemical sensing [17], and biomedicine [18].

Among the various ZIFs, ZIF-8 is one of the most widely investigated, having a sodalite (SOD) topology and displaying a three-dimensional structure composed of zinc ions and 2-methylimidazole (2-MeIM) linkers. ZIF-8 has large pores, with diameters of 1.16 nm, which are accessible through small apertures with diameters of 0.34 nm, and they have a cubic space group with unit cell dimensions of 1.632 nm [19]. ZIF-8 possesses high thermal stability to 550 °C in N_2_, a large surface area (1630–1700 m^2^/g), and exceptional chemical resistance to various solvents [20]. The crystal sizes of ZIF-8 depend on several synthesis conditions. ZIF-8 can be prepared using several different synthesis routines in water or organic solvents, such as conventional solvothermal [21] or hydrothermal [22], colloidal precipitation [23,24], microwave-assisted [25], mechanochemical [25], sonochemical [25], dry-gel [25], electrochemical [26], microfluidic [25], or template [27] methods. The hydrothermal or solvothermal processes are generally conducted with a reaction temperature from room temperature up to 200 °C and a reaction duration from hours to days. Banerjee et al. [21] found that a metal precursor concentration of 0.20 M, a temperature of 85 or 100 °C, and a reaction time of 72 h were optimal conditions for preparing ZIF nanoparticles. The colloidal precipitation method is a facile route [23] for the mass production of nano- to micro-meter scale ZIF-8 crystals with controlled particle sizes, and has been widely used in the literature.

Mphuthi et al. [16] reported that the average powder size of ZIF-8 was 65 nm (synthesized in water) and 52 nm (prepared in methanol). Zheng et al. [28] found that ZIF-8 crystals with sizes of 40~ 110 nm could be obtained in microemulsions, by controlling the ratio of Zn^2+^ to 2-MeIM at 1:16~1:2. Venna et al. [19] found that the structural evolution of ZIF-8 is a function of synthesis time, which can be divided into three stages: (I) the nucleation stage (*t* < 10 min); (II) the growth stage (a maximum in crystallinity is achieved at ~60 min); and (III) the stationary phase (*t* > 60 min). The relative crystallinity of ZIF-8 increased slowly in the initial stage of synthesis. Next, a rapid increase was reported in the 30–40 min range. The relative crystallinity of ZIF-8 was almost unchanged after 50 min (above 90%), and the relative crystallinity of ZIF-8 reached a maximum at 24 h [19]. Different zinc salts with a methanol-based recipe have been reported to have different effects on the crystal sizes of ZIF-8. It has been observed that greater nucleation rates led to smaller crystal sizes for zinc nitrate, while the reverse was true for ZIF-8 crystals from zinc acetate [23,29]. Ramu et al. [30] found that zinc nitrate was a more effective zinc source, probably due to the fast nucleation of zinc nitrate compared to other zinc salts.

In order to improve the chemical stability of ZIF-8, nitrogen-functionalized and Zn-decorated microporous carbon materials can be obtained via the carbonization of ZIF-8 with imidazolate framework ligands and Zn metal centers (melting point of 419.53 °C and boiling point of 907 °C) [31]. Torad et al. [23] synthesized nanoporous carbon by the direct carbonization of ZIF-8 crystals, and observed that the products showed a large adsorption uptake and a faster sensor response for toluene vapors. A systematic study was conducted by Gadipelli and Guo [31], where the carbonization process of ZIF-8 was reported. The results showed that the ligand decomposition and metal evaporation were highly associated with the carbonization temperature, reaction time, and heating rate. As the temperature was above 800 °C, this would result in the development of porosity at the expense of Zn and N atoms, as well as the yield of the products. The mass loss could be attributed to the dissociation of -CH_3_ groups (commencing at approximately 600 °C), the ligand decomposition and liberation of –C-N-H groups (between 600 and 900 °C), and the evaporation of Zn metal (above 900 °C). Nevertheless, the actual framework decomposition and subsequent carbonization were relatively slow compared with the rapid decomposition of other MOF structures with carboxylate ligands. When ZIF-8 was heat treated at a temperature of 1000 °C, nitrogen-doped carbon nanopolyhedra were obtained with a highly graphitic carbon skeleton, during which Zn ions in ZIF-8 were reduced to Zn metal and vaporized. The abundant nitrogen in ZIF-8 was directly incorporated into an aromatic methyl-imidazole ligand, which could promote the combination of enriched nitrogen-containing active sites into carbon structures. The resultant carbons had a hierarchical porous structure and uniform distribution of active sites [32]. Therefore, the carbonization of ZIF-8 at different temperatures (from 600 to 1100 °C) yielded different carbon structures.

MOF-derived nanoporous carbons have been used for electrochemical double-layered capacitors; though they had a high specific surface area, their many micropores led to poor conductivity and resulted in a weak capacitance [33,34]. Two Zr-based MOFs were doped into the polyimide-based nanofibers to form nanocomposites, and these nanocomposites could remove non-polar and polar volatile organic compounds from the air. The results showed that the introduction of MOFs powders significantly improved the adsorption performance of the nanofibers [35]. MOFs can be synthesized in situ on electrospun PAN-based nanofibers using two processes: electrospinning and aqueous synthesis, where MOFs/PAN composites served as the SO_2_ gas sensing layer. The sensors comprised MOFs/PAN composites, and carbon nanotubes possessed remarkable sensitivity and long-term stability to SO_2_ gas [36]. Wang et al. [37] reported that when ZIF-67 was cast on carbon cloth and then polyaniline was electrodeposited on composites, they exhibited a good capacitance. A ZIF-based carbon fiber for supercapacitors was prepared via the pyrolysis of ZIF-8-based polyacrylonitrile (ZIF-8/PAN) nanofibers, where ZIF-8: PAN precursor = 3:4 (*w/w*). In this study, after ZIF-8 was carbonized at 800 °C for 3 h, the Zn totally disappeared in the ZIF-8/PAN fibers [38].

Wang et al. [39] utilized an in situ growth method, composed of electrospinning and a hydrothermal treatment, to prepare a ZIF-8/PAN fibrous filter, and reported that U(VI) chelated with the N atoms of 2-MeIM was the major adsorption mechanism, while in situ ZIF-8/PAN fibers exhibited a high adsorption capacity for applications to nuclear wastewater. The in situ growth method was used to prepare ZIF-8/cellulose nanofiber composites [40]. Due to the electrostatic forces between Zn metal in ZIF-8 and –COO- groups on nanofibers, ZIF-8 crystals could be homogenously deposited on nanofibers. The composites showed good CO_2_/N_2_ selectivity. A novel structure of PAN nanofibers was fabricated using an electrospinning technique, decorated with ZIF-8 using a phase conversion growth method, and investigated for CO_2_ capture [41]. The results indicated that electrostatic interaction and hydrogen bonds existed between the PAN matrix and the ZIF-8 nanocrystals confined to the PAN nanofiber surface. However, the CO_2_ uptakes were only 7.0 cm^3^/g at 25 °C, 1 bar. Recently, a methodology was proposed to predict the purity, yield, and crystallinity of ZIF-8 crystals prepared via electrochemical routes, and which used the application of artificial intelligence, involving the design of experiments with a machine learning module [42]. This could be a feasible route towards the sustainable design and development of advanced materials and chemicals. Before it is practically applied, the collection of baseline information is important.

**Scheme 1:**
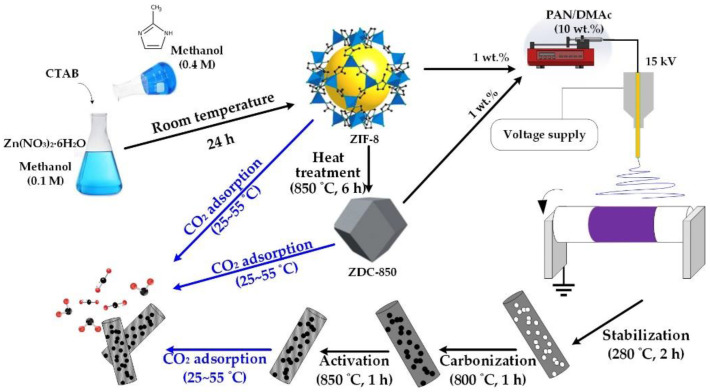
Schematic illustration of this study.

Several synthesis techniques or novel structures have been reported for the growing applications of ZIF-8. ZIF-8-based nanomaterials were selected because they are one of the most investigated MOFs and as they have various applications, especially based on adsorption. The interactions between CO_2_ and ZIF-8 can enhance the storage of CO_2_ in the framework, and the storage performance of CO_2_ on ZIF-8 was strongly pressure dependent [43]. At present, the CO_2_ adsorption capacities of pristine ZIFs crystals were not distinct enough at, or under, atmospheric pressure [6,41,44]. Thus, several studies were focused on applications with high pressure [14,43]. Most studies on ZIFs have focused on the selectivity of CO_2_ from various gas mixtures [6,8,45]. However, currently, studies on the high-temperature activation of ZIF/PAN nanocomposites have not been published in the literature. Here, for the first time, PAN-based activated nanofiber composites embedded with ZIF-8 crystals or ZIF-8-derived carbons (ZDC) were fabricated using successive processes, comprising electrospinning, stabilization, carbonization, and activation. The properties of the adsorbents were characterized thoroughly, and the CO_2_ adsorption performance of the adsorbents was investigated. It was intended to incorporate the advantages of carbon nanofibers with the benefits of ZIF-8 based nanoparticles, to develop high-performance CO_2_ adsorbents. A schematic diagram of this study is shown in Scheme 1.

## 2. Materials and Methods

### 2.1. Chemicals

Zinc nitrate hexahydrate (≥99%) was purchased from J.T. Baker (Radnor, PA, USA); 2-methylimidazole (2-MeIM, 99%) and polyacrylonitrile (PAN) (Mw = 150 kDa) were obtained from Sigma-Aldrich (St. Louis, MO, USA); cetyltrimethylammonium bromide (CTAB, 98%) and N, N-dimethylacetamide (DMAc, 99%) were purchased from Alfa Aesar (Sisli-Istanbul, Turkey); methanol (≥99.8%) was purchased from Mallinckrodt Chemicals (Phillipsburg, NJ, USA). All the chemicals were used without further purification.

### 2.2. Preparation of ZIF-8

A rapid colloidal chemistry route was used to produce ZIF-8 nanocrystals at room temperature [24,46]. The sizes of nanocrystals were highly related to the molar ratio of 2-MeIM to zinc salt [28]. In a typical process, 0.74 g of zinc nitrate hexahydrate and 19 mg CTAB were dissolved in 25 mL methanol, and 0.82 g of 2-MeIM was dissolved in 25 mL methanol, respectively. CTAB was used as a surfactant. Additionally, the methanol solution of zinc nitrate hexahydrate was quickly poured into the methanol solution of 2-MeIM under stirring, at room temperature. After rigorous and continuous stirring for 30 min, the mixture solution was placed at room temperature to settle for 24 h [19]. Finally, the white precipitate was collected and thoroughly washed with methanol using centrifugation to remove the excess 2-MeIM. The ZIF-8 powders were obtained after drying at 80 °C for 24 h in an oven.

### 2.3. Heat Treatment of ZIF-8

In order to improve the chemical stability of ZIF-8, carbonization of ZIF-8 powders was conducted. In a typical carbonization process, 0.5 g of ZIF-8 was placed in a porcelain combustion boat and then transferred into a tubular furnace. The samples were heated in a nitrogen atmosphere with a heating rate of 5 °C/min, and carbonized at 850 °C for 6 h. The sample was named ZDC-850.

### 2.4. Fabrication of Electrospun Nanofiber Composites

Nanofiber composites were prepared using an electrospinning process. Initially, PAN was dissolved in DMAc to form a polymer solution with 10 wt.% PAN. The resulting solutions were subjected to continuous magnetic stirring at 60 °C for 24 h in order to obtain a homogeneous polymer solution. Next, ZIF-8 (or ZDC-850) with a ratio of 10 wt.% of ZIF-8 (or ZDC-800) to PAN was added into the polymer solution and continuously stirred for 24 h at room temperature. Electrospinning equipment (FES-COE, Falco Tech Enterprise Co., Ltd., Taiwan) was used for fiber electrospinning. The electrospinning was carried out under an applied electrical voltage of 15 kV onto a metal drum collector (∅ 15 cm) rotated at 300 rpm and covered with aluminum foil, and a tip-to-collector distance of 15 cm was used. The mixture solution was pumped at a flow rate of 1.0 mL/h, pushed by a syringe pump (NE-1000, New Era Pump Systems, Inc., Farmingdale, NY, USA). A 21-gauge needle (inner diameter: 0.52 mm) was selected.

The electrospun fibers were subjected to the following post-treatments. First, the fibers were stabilized in air, from room temperature to 280 °C at a heating rate of 1 °C/min; then, the temperature was held at 280 °C for 2 h to complete the cyclization and dehydrogenation reactions for converting PAN from a thermoplastic to a non-plastic compound [47,48,49]. The stabilized nanofibers were cooled down to room temperature and then carbonized at 800 °C with a heating rate of 5 °C/min, and maintained for 1 h, before the sample was cooled down to room temperature, in a nitrogen atmosphere with a flow of 100 sccm in a tubular furnace. After carbonization, an activation process was conducted by raising the temperature to 850 °C at a rate of 10 °C/min under flowing nitrogen. Then, CO_2_ was switched in and held for 1 h, which was used as the activation agent with a flow rate of 100 sccm. The product was denoted as ZIF-8/PAN (or ZDC-850/PAN). For comparison, the pure PAN carbon nanofibers were prepared and labeled as PAN.

### 2.5. Characterizations

Field emission scanning electron microscopy (FESEM) with a microscope (S-4700, Hitachi, Krefeld, Germany) was utilized to probe the morphology of the samples. High-resolution transmission electron microscopy (HRTEM) images were obtained using a Philips/FEI Tecnai 20 G2 S-Twin Transmission Electron* Microscope (Hillsboro, OR, USA), to observe the morphology of the distribution of Zn metals deposited in the nanofiber composites. X-ray diffraction (XRD) was performed using a powder diffractometer (Rigaku TTRAX III, Tokyo, Japan) equipped with Cu-Kα radiation (λ = 0.15418 nm) at 30 kV and 20 mA. The XRD patterns were collected over 5–90° in 2θ mode at a rate speed of 4°/min. Thermogravimetric analysis (TGA) was used to determine the thermal stability of the samples in flowing nitrogen (60 cm^3^/min) with a heating rate of 10 °C/min. A thermogravimetric analyzer (Dynamic TGA Q500 in TA Instrument 5100) (TA Instrument, New Castle, DE, USA) was employed to measure any changes in the weight of the sample, as a function of temperature.

The surface properties of the samples were estimated from N_2_ adsorption–desorption isotherms measured at −196 °C, using an ASAP 2020 accelerated surface area and porosimetry system (Micromeritics, Norcross, GA, USA). Prior to the adsorption measurements, the samples were outgassed at 350 °C for 24 h. One exception was ZIF-8, which was degassed at 90 °C for 24 h, to prevent changes in the structure. The specific surface areas (SSAs) of the samples were measured in P/P_o_ = 0.05~0.3 using the Brunauer–Emmett–Teller (BET) method. The micropore (<2.0 nm) surface area (S_mi_) was determined using the t-plot method. The single point total pore volume (V_t_) was obtained at P/P_o_ ≈ 0.99. The mesopore volume (V_me_), micropore volume (V_mi_), and ultra-micropore (<0.7 nm) volume (V_<0.7nm_) were obtained by applying a non-local density functional theory (NLDFT) model, where V_<0.7nm_ is part of V_mi_. Pore size distribution curves were also acquired with an NLDFT model. Elemental analysis (EA) was used to analyze the elemental compositions in the samples, where the weight percent (wt.%) of the C, H, and N elements was measured using an elemental analyzer (Elementar vario EL cube, Langenselbold, Germany). The chemical states on the surface of the samples were determined by X-ray photoelectron spectroscopy (XPS). The XPS spectra were collected using a spectrophotometer (PHI 5000 VersaProbe II, ULVAC-PHI, Kanagawa, Japan), in which a scanning X-ray monochromator (Al Anode, hν = 1401 eV) was used, and information on the elements within a few nanometers of the sample surface could be acquired. For calibration purposes, the C 1s electron binding energy (B.E.) that corresponds to graphitic carbon was set at 285 eV. The deconvolution of the XPS spectra was carried out using a nonlinear least squares curve-fitting program (XPSPEAK software, version 4.1, The Chinese University of Hong Kong, Hong Kong, China).

### 2.6. CO_2_ Adsorption Experiments

The CO_2_ adsorption isotherms on the samples were measured using the Micromeritics ASAP 2020 system. The samples were degassed at 350 °C for 24 h for removal of adsorbed contaminants prior to the measurement. For ZIF-8, the degas process was conducted at 90 °C for 24 h. The equilibration time for each pressure point was 45 s. An ISO Controller (Micromeritics), a thermoelectric cooled dewar, was utilized for temperature control during the CO_2_ adsorption process.

Based on our previous studies [50,51], the Freundlich equation [52] was used for adsorption data fitting. The Freundlich adsorption isotherm (Equation (1)), an empirical model, assumes heterogeneous adsorption, due to the diversity of adsorption active sites:

(1) $$q_{e}=K_{F}P^{1/n},$$

where $q_{e}$ (mmol/g) is the equilibrium adsorption capacity, $K_{F}$ [(mmol/g)(1/kPa)^1/*n*^] is the Freundlich adsorption coefficient, *P* (kPa) is the gas pressure, and *n* is a constant indicating the isotherm curvature.

When adsorbate molecules are adsorbed from the bulk gas phase to the adsorbed phase, the change in enthalpy can be measured by the isosteric heat of adsorption ($Q_{st}$) [52]. $Q_{st}$ denotes the interactions between the gas molecules and the adsorbent lattice atoms and provides a measure of energetic heterogeneity to the gas–solid interfaces [53]. The Clausius–Clapeyron equation (Equation (2)) was used to determine the values of $Q_{st}$:

(2) $$-\frac{Q_{st}}{R}=\left(\frac{d\ln P}{d\;\frac{1}{T}}\right),$$

where $Q_{st}$ (kJ/mol) is the isosteric heat of adsorption, *R* (= 8.314 J/mol/K) is the gas constant, *P* (kPa) is the CO_2_ pressure, and *T* (K) is the adsorption temperature.

## 3. Results and Discussion

### 3.1. Field Emission Scanning Electron Microscopy (FESEM) Images

**Figure 1:**
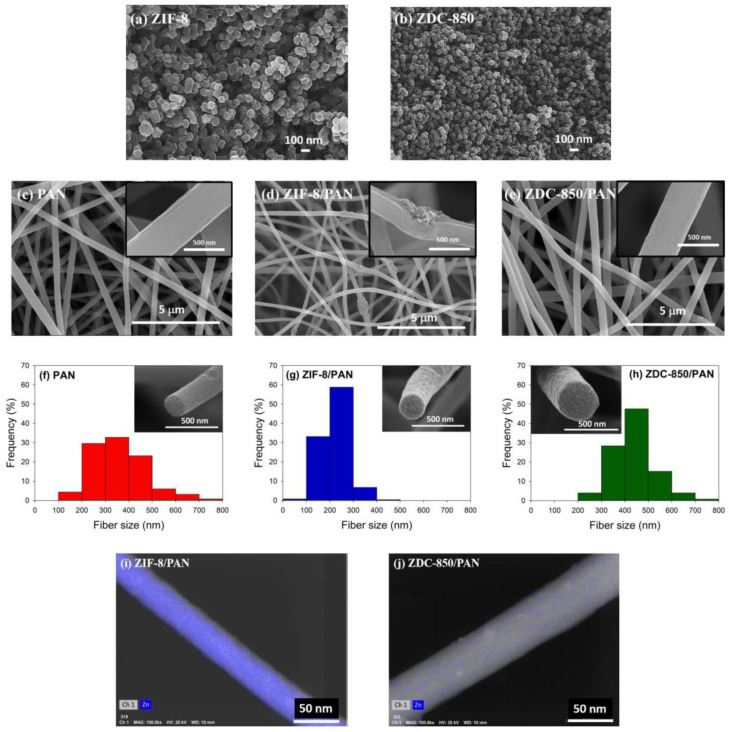
FESEM images, fiber size distributions, and the HRTEM-EDX mapping of the samples: (**a**) ZIF-8; (**b**) ZDC-850; (**c**,**f**) PAN; (**d**,**g**,**i**) ZIF-8/PAN; (**e**,**h**,**j**) ZDC-850/PAN.

Figure 1a shows the FESEM images of ZIF-8 crystals prepared in this study, which were uniform in size, with an average of approximately 63 nm. An excess of 2-MeIM ligands and CTAB (surfactants) shielded the particles from further growth, leading to the formation of nanoparticles [16]. The carbonization of ZIF-8 at 850 °C for 6 h resulted in particles with a mean size reduced to 46 nm (Figure 1b). Moreover, the degree of crystallinity was also deteriorated after carbonization. Figure 1c–e show the FESEM images of PAN, ZIF-8/PAN, and ZDC850-PAN, in which the insets are the images of a single fiber. It could be observed that the average diameter of PAN nanofibers was approximately 350 nm (Figure 1f). When introducing ZIF-8 in PAN precursor solution, the fiber diameter of the fabricated composites became smaller (~220 nm) (Figure 1g), but some nodes appeared in the fibers. If incorporated with ZDC-850, the generated fibers almost remained smooth and straight, with a diameter of approximately 430 nm (Figure 1h). It is supposed that the ZIF-8 crystals in the PAN fibers would act as active sites. The ligand decomposition of ZIF-8 in the fibers in the subsequent carbonization and activation processes could promote the volatilization of low-molecular-weight segments in the PAN fibers. On the other hand, the incorporation of ZDC-850 into the PAN fibers did not show this phenomenon; thus, leading to larger fiber diameters. Figure 1i,j shows HRTEM-EDX mapping of ZIF-8/PAN and ZDC-850/PAN, where the Zn element was equally spread on the surface of the nanofibers.

### 3.2. X-ray Diffraction (XRD) Patterns

**Figure 2:**
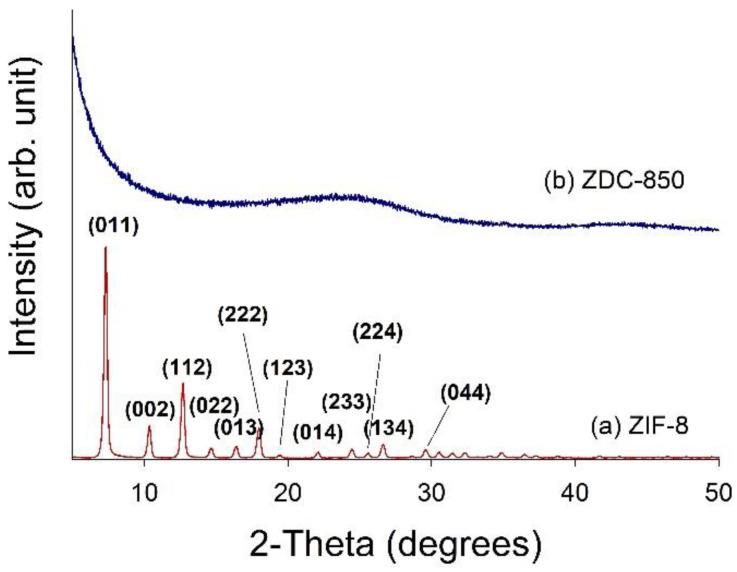
The XRD patterns of ZIF-8 and ZDC-850.

The XRD patterns of ZIF-8 and ZDC-850 are shown in Figure 2. The typical characteristic peaks at 2θ of 7.3, 10.4, 12.7, 14.6, 16.4, 18.0, 19.5, 22.1, 24.5, 25.6, 26.6, and 29.6° are ascribed to the (011), (002), (112), (022), (013), (222), (123), (114), (233), (224), (134), and (044) planes of ZIF-8 (CCDC 602542, [41]), respectively. However, the framework decomposition was observed in the XRD pattern of ZDC-850, where only one detectable peak was observed at approximately 2θ = 26°, belonging to a typical (002) interlayer peak of graphite-type carbons. The XRD results were consistent with the FESEM images.

### 3.3. Thermogravimetric Analysis (TGA) Profiles

**Figure 3:**
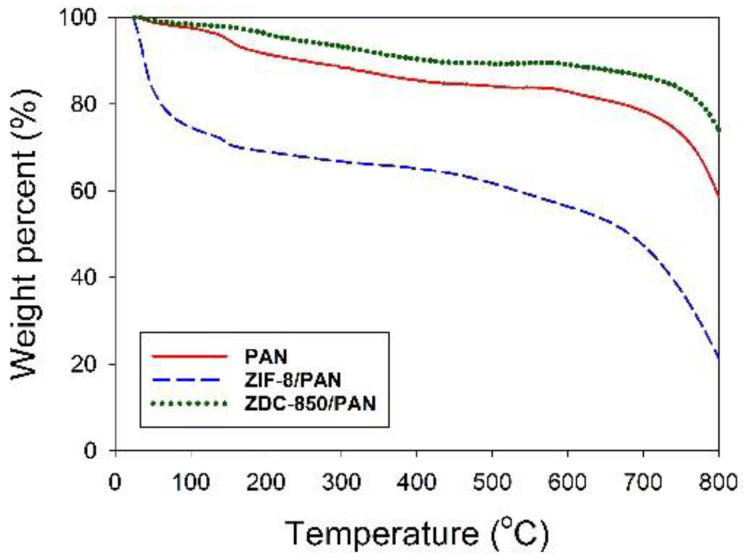
The TGA patterns of PAN, ZIF-8/PAN, and ZDC-850/PAN.

Figure 3 displays the TGA profiles of PAN, ZIF-8/PAN, and ZDC-850/PAN samples in a nitrogen atmosphere. Approximately 60% of PAN and 70% of ZDC-850/PAN remained when the samples were heated to 800 °C under a nitrogen atmosphere. On the other hand, the ZIF-8/PAN composites had a rapid weight loss before 100 °C, but the weight was stable up to approximately 500 °C under flowing nitrogen, and approximately 20% of the ZIF-8/PAN remained after it was heat treated at 800 °C under a nitrogen atmosphere. The TGA profiles illustrate that the embedding of ZDC-850 into PAN-based carbon nanofibers could enhance the thermal stability of the carbon nanofibers. However, the introduction of ZIF-8 in nanofibers decreased the stability.

### 3.4. ASAP Data

**Figure 4:**
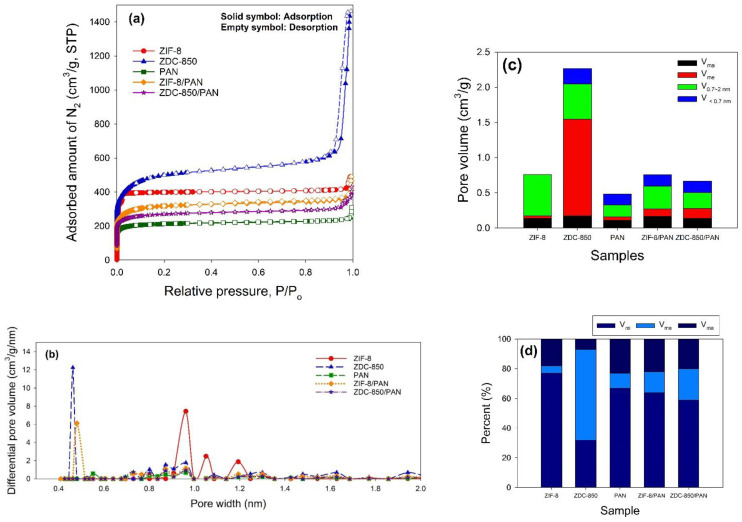
Adsorption–desorption measurements of N_2_ for the samples at −196 °C: (**a**) isotherms; (**b**) pore size distributions of the samples, which were derived using NLDFT model; (**c**) distributions of different pore volumes; (**d**) percent of pore volumes.

The N_2_ adsorption–desorption isotherms of most samples at −196 °C (Figure 4a) belonged to type I according to the Brunauer–Emmett–Teller (BET) classification. There were no hysteresis loops on these isotherms, indicating that the samples were microporous. One exception was ZDC-850. It exhibited a type IV adsorption isotherm with a distinct hysteresis loop at a relative pressure higher than 0.8. This hysteresis loop was identified as H1, according to the International Union of Pure and Applied Chemistry (IUPAC) classification, implying that the samples were agglomerates of spheroidal particles, of fairly uniform size. Figure 4b shows the pore size distributions in the range of 0.4–2 nm, which were derived from the heterogeneous surface, two-dimensional non-local density functional theory (HS-2D-NLDFT) model. ZIF-8 had significant pore volumes in the range of 0.9–1.3 nm, while ZDC-850 and ZIF-8/PAN exhibited a distinct peak at approximately 0.5 nm. The pores with sizes less than 0.7 nm are labeled as ultra-micropores, which are considered as an important feature affecting CO_2_ uptake [1,2,3,4,5]. Figure 4c displays the pore volumes in different size ranges, including the volumes with pore sizes less than 0.7 nm, 0.7–2 nm, 2–50 nm (V_me_), and greater than or equal to 50 nm (V_ma_). Figure 4d presents the percent of pore volumes classified as macropores, mesopores, and micropores. The pore volume distributions indicated that the mesopores were predominant on ZDC-850, but micropores were primarily found on the other materials. These results verified the fact that the hysteresis loop in the isotherm of ZDC-850 was ascribed to the capillary condensation occurring in mesopores.

The SSAs of ZIF-8 and ZDC-850 were 1579 and 1873 m^2^/g, respectively. The SSAs of the composites, i.e., the nanofibers containing 10 wt.% ZIF-8 or ZDC-850, were increased compared with the pure PAN, where the SSAs of PAN, ZIF-8/PAN, and ZDC-850/PAN were 840, 1178, and 1046 m^2^/g, respectively. The ligand decomposition of the individual ZIF-8 crystals or in the PAN nanofibers at high temperatures facilitated the increase in specific surface areas and pore volumes. The micropore area (S_mi_) were 1525 (ZIF-8), 1191 (ZDC-850), 716 (PAN), 904 (ZIF-8/PAN), 842 (ZDC-850/PAN) m^2^/g, respectively. As seen from the data, ZIF-8 and PAN had higher S_mi_/SSA and V_mi_/V_t_ ratios, while PAN and ZDC-850/PAN exhibited higher V_<0.7nm_/V_t_ ratios.

### 3.5. Elemental Analysis (EA)

**Figure 5:**
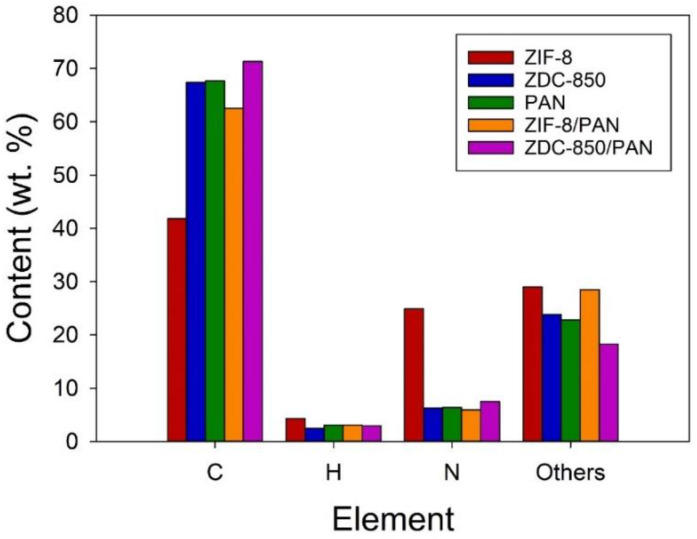
The weight percentage of the elements in the bulk phase of the samples obtained from the elemental analysis (EA).

Figure 5 presents the elemental compositions of C, H, and N atoms in the bulk phase of the samples. An abundant nitrogen content was measured in ZIF-8 (24.9 wt.%), which could be attributed to the fact that ZIFs are made up of nitrogen-rich ligands. The PAN nanofibers contained a certain amount of nitrogen, remaining from the PAN precursor. It is thought that the ZIF-8 crystals introduced into PAN nanofibers were reactive at the carbonization and activation temperatures; hence, the subsequent processes could lead to the loss of some nitrogen. Since ZDC-850 was relatively more stable than ZIF-8, the incorporation of ZDC-850 into PAN fibers could instead increase the nitrogen content.

### 3.6. X-ray Photoelectron Spectroscopy (XPS)

**Figure 6:**
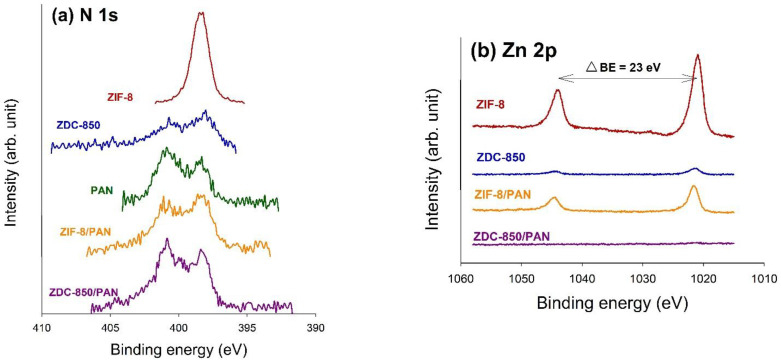
The X-ray photoelectron spectroscopy (XPS) profiles of the samples: (**a**) N 1s peaks; (**b**) Zn 2p peaks.

An XPS analysis was performed to reveal the chemical compositions, oxidation states, and atomic ratios of the elements on the surface of the samples. The XPS spectra indicated the existence of C 1s, N 1s, O 1s, and Zn 2p on the surface of the samples. Their atomic ratios are comparatively summarized in Table 1. Abundant amounts of N 1s (26.62 at.%) and Zn 2p (13.91 at.%) were measured on ZIF-8, which signified the feature of imidazolate-like ligands and Zn metals. Nevertheless, the contents of N 1s and Zn 2p decreased obviously when the samples were subjected to heat treatment. The peaks of Zn 2p even disappeared on ZDC-850/PAN. The XPS regions of N 1s and Zn 2p are shown in Figure 6. The N 1s peak of ZIF-8 showed a unimodal symmetrical spectrum, while bimodal spectra were observed on ZDC-850 or the nanofiber composites, implying different functional group distributions. The Zn 2p_3/2_ photoelectron line had binding energies of approximately 1021 eV, with a spin-orbit splitting of 23 eV between the Zn 2p_3/2_ and Zn 2p_1/2_. The atomic ratios of Zn 2p in ZIF-8, ZDC-850, and ZIF-8/PAN were 13.91, 0.84, and 1.29, respectively. When ZIF-8 crystals were incorporated into the PAN fibers, the Zn metal could be embedded and protected by the PAN fibers from excess volatilization in the following treatments, although sometimes they might be exposed, as shown in the inset of Figure 1d. For ZDC-850/PAN, although Zn was not detected by XPS, trace Zn was observed using EDX. The weight percentage of Zn was 35.33 (ZIF-8), 2.90 (ZDC-850), 13.82 (ZIF-8/PAN), and 3.86 (ZDC-850/PAN). This demonstrated that ZDC-850 was embedded inside, not on the surface.

High-resolution XPS N1s spectra can be deconvoluted into four functional groups at 398.4, 400.5, 401.2, and 404.6 eV [54,55,56,57]; these can be assigned to N=C, O=C-NH, quaternary or protonated N, and oxidized species, respectively. The calculated atomic ratios and relative percentages of the functional nitrogen atoms are displayed in Table 2. ZIF-8 had only two N-containing groups, i.e., N=C and O=C-NH, which were ascribed to the imidazolate-like ligands of the ZIF-8 building blocks. After ZIF-8 was carbonized at 850 °C, certain amounts of oxidized N species would be generated. On ZIF-8/PAN, the introduced ZIF-8 could contribute N=C groups. For ZDC-850/PAN, the doping of ZDC-850 primarily increased the quaternary or protonated N groups. The atomic ratios of N 1s on all samples are also shown in Table 2, which are in agreement with the results of the EA analysis; implying that the samples were homogeneous for N-distribution.

Table 3 summarizes the deconvolution results of the XPS O 1s spectra. The curve fitting disclosed the presence of three oxygen-containing functional groups [58,59,60], namely, the C=O/O=C-N (531.5 eV), C-OH (532.7 eV), and COOH (533.7 eV) groups. The C=O/O=C-N groups were the only type of surface oxides on ZIF-8, implying that ZIF-8 was almost oxygen-free. One extra surface oxide, -COOH, was measured on ZDC-850. In addition, the introduction of ZIF-8 or ZDC-850 into PAN nanofibers could decrease the oxygen content. For all of them, the C=O/O=C-N groups were primarily oxygen functional groups.

High-resolution XPS C1s regions can be deconvoluted into five functional groups: due to aliphatic and aromatic backbones (C-C/C=C) at 285 eV and carbon atoms present in C-N (286.1 eV), C=N/C-OH (286.6 eV), C=O (287.6 eV), and COOH (290.5 eV) [54,58,59,61]. Table 4 displays the atomic ratios and relative percentages of non-functional and functional carbon atoms. For all samples, the predominant groups were the C-C and C=C groups. In particular, ZDC-850/PAN had a large amount of non-functional carbon groups. It should be noted that ZIF-8/PAN possessed a significant amount of C-N groups, and ZDC-850 was in second place.

### 3.7. CO_2_ Adsorption Performance

**Figure 7:**
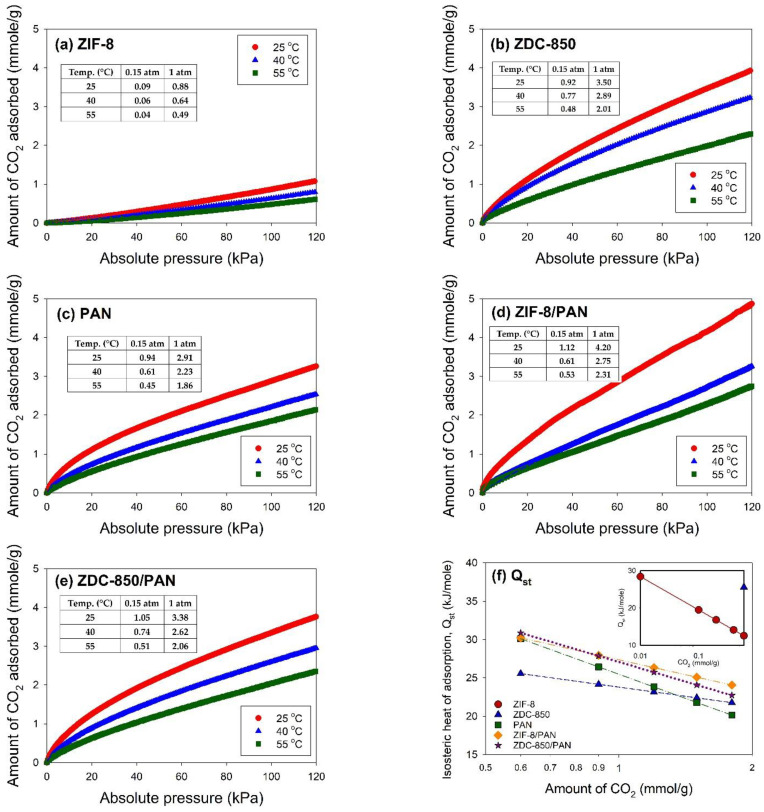
Adsorption isotherms of CO_2_ of the samples: (**a**) ZIF-8; (**b**) ZDC-850; (**c**) PAN; (**d**) ZIF-8/PAN; (**e**) ZDC-850/PAN; and (**f**) the isosteric heat of adsorption of CO_2_ on the samples.

The CO_2_ adsorption isotherms for all samples under a pressure of less than 123 kPa at 25, 40, or 55 °C are shown in Figure 7. An equilibrium duration of 45 s was maintained for each measurement point, after achieving the set pressure value. The CO_2_ uptake increased with increasing CO_2_ pressure, but decreased with increasing adsorption temperature, manifesting the characters of the exothermic reactions. As seen from Figure 7a, the CO_2_ uptakes of the as-prepared ZIF-8 seemed not to behave well under atmospheric pressure. The CO_2_ adsorption capacities at 25 °C and 1 atm followed the order: ZIF-8/PAN (4.20 mmol/g) > ZDC-850 (3.50 mmol/g) > ZDC-850/PAN (3.38 mmol/g) > PAN (2.91 mmol/g) > ZIF-8 (0.88 mmol/g). At 25 °C and 0.15 atm (a typical untreated flue gas composition), the CO_2_ uptakes had a different order: i.e., ZIF-8/PAN (1.12 mmol/g) > ZDC-850/PAN (1.05 mmol/g) > PAN (0.94 mmol/g) > ZDC-850 (0.92 mmol/g) > ZIF-8 (0.09 mmol/g).

**Figure 8:**
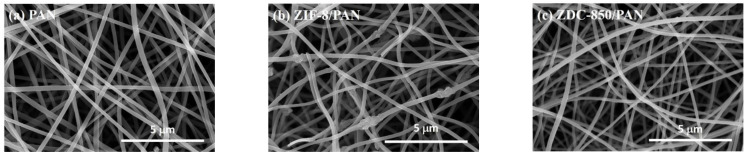
FESEM images of (**a**) PAN, (**b**) ZIF-8/PAN, and (**c**) ZDC-850/PAN after CO_2_ adsorption.

At 25 °C and 1 atm, ZIF-derived carbons and ZIF-related nanofiber composites exhibited a far superior CO_2_ adsorption performance than the as-prepared ZIF-8 or pure PAN nanofibers. However, in the lower pressure (0.15 atm), the nanofibers, namely, PAN, ZIF-8/PAN, and ZDC-850/PAN, displayed better CO_2_ adsorption capacities than ZIF-8 or ZDC-850. These findings indicate that the specific surface areas and total pore volumes of the adsorbents may not be crucial parameters for CO_2_ capture. The comprehensive interactions between hierarchical porous structures, surface functional groups, and the Zn metal active sites dominated the CO_2_ adsorption performance. Under 1 atm at 25 °C, the Zn metal active sites inside the pores, the pores at approximately 0.5 nm, and C-N (amines) groups could promote CO_2_ adsorption. At low pressures, the carbon content in the adsorbents should be higher than a threshold. Under this condition, the percentage of ultra-micropore and micropore volumes, as well as the functional groups, such as the quaternary or protonated N (amines), N=C (imines or pyridine-type N), C-OH, and -COOH groups, should be considered highly important. The imines and amines groups are usually regarded as the Lewis base and expected to generate distinct interactions with CO_2_ molecules (as a weak Lewis acidic gas) [4,62,63,64]. Figure 8 shows FESEM images of PAN, ZIF-8/PAN, and ZDC-850/PAN, which underwent the CO_2_ adsorption processes. Compared with the images in Figure 1, this demonstrates that the samples could maintain their robustness after CO_2_ adsorption.

The Freundlich equation was used for the curve-fitting of the CO_2_ measurement data. The consequences are summarized in Table 5 and also displayed in Figure 7a–e. As seen from the data, the values of $K_{F}$ varied inversely, proportionally to the adsorption temperature. The values of *n* ranged from 0.69 to 1.65 and were indicative of a feasible adsorption, except for ZIF-8. The Q_st_ values varied with the CO_2_ uptakes, as shown in Figure 7f, in which the Q_st_ values log-linearly decreased with the CO_2_ loading, from 0.6 to 2 mmol/g, indicating that the adsorption active sites on the surface of the adsorbents were energetically heterogeneous for CO_2_ capture [65]. The data of ZIF-8 are shown in the inset of Figure 7f, due to its low adsorption capacities. Based on the Q_st_ values, which were lower than 40 kJ/mol, we concluded that the CO_2_ adsorption on the adsorbents belonged to a typical physical adsorption process. The Q_st_ values had a lower CO_2_ loading response to the CO_2_ adsorption performance at low CO_2_ partial pressures, indicating the interactions between CO_2_ molecules and the surface functional groups or the enhanced micropore confinement [66]. The slope in the log–linear plot was highly related to their CO_2_ adsorption capacities. Table 6 summarizes the CO_2_ uptakes of the adsorbents used in this study and various ZIF-8-related adsorbents in the literature, which implied that the CO_2_ adsorption on the ZIF-8/PAN, ZDC-850, and ZDC850-PAN prepared in this study performed well for CO uptake.

## 4. Conclusions

This study successfully fabricated ZIF-8, ZIF-8-derived carbon (ZDC-850), and their activated PAN-based nanofiber composites. Due to the ligand decomposition, and partly due to the evaporation of Zn metal in ZDC-850, the particle sizes of ZDC-850 were smaller than those of the as-prepared ZIF-8. The degree of crystallinity of the ZDC-850 totally changed compared to that of ZIF-8. The properties of nanofiber composites depend on the dopants (ZIF-8 or ZDC-850); nevertheless, both could increase in surface area and pore volume. ZDC-850 was mesoporous, while the others were microporous. ZIF-8 had significant pore volumes in the range of 0.9–1.3 nm, but ZDC-850 and ZIF-8/PAN exhibited a distinct peak at approximately 0.5 nm. The Zn metals in the ZIF-8/PAN or ZDC-850/PAN could be embedded and protected by the PAN fibers from excess volatilization in the following treatments: At 25 °C and 1 atm, ZIF-derived carbons (3.50 mmol/g) and ZIF-related nanofiber composites (4.20 mmol/g for ZIF-8/PAN, 3.38 mmol/g for ZDC-850/PAN) exhibited far superior CO_2_ adsorption performance than the as-prepared ZIF-8 (0.88 mmol/g) or pure PAN nanofibers (2.91 mmol/g). However, at lower pressure (0.15 atm), the nanofibers, namely, PAN, ZIF-8/PAN and ZDC-850/PAN, displayed better CO_2_ adsorption capacities than ZIF-8 or ZDC-850. These findings demonstrate that the specific surface areas and total pore volumes of the adsorbents may not be crucial parameters for CO_2_ capture. The comprehensive interactions between hierarchical porous structures, surface functional groups, and the Zn metal active sites dominated the CO_2_ adsorption performance.

## Figures and Tables

**Table 1:** The atomic ratios (at.%) of surface elements from the X-ray photoelectron spectroscopy (XPS) profiles.

Sample	C 1s	N 1s	O 1s	Zn 2p
ZIF-8	56.25	26.62	3.21	13.91
ZDC-850	90.73	4.06	4.37	0.84
PAN	86.58	4.52	8.90	0.00
ZIF-8/PAN	87.78	4.52	6.42	1.29
ZDC-850/PAN	87.55	4.96	7.50	0.00

**Table 2:** Results of the fit of the XPS N 1s region; values given in at.% (percentage of total intensity).

Binding Energy (eV)	Functional Group	ZIF-8	ZDC-850	PAN	ZIF-8/PAN	ZDC-850/PAN
398.4	N=C	25.96	1.85	2.50	2.62	2.24
		(97.50%)	(45.69%)	(55.22%)	(58.07%)	(45.21%)
400.5	O=C-NH	0.66	0.19	0.64	0.26	0.56
		(2.50%)	(4.59%)	(14.08%)	(5.73%)	(11.31%)
401.2	Quaternary or protonated N	—	0.85	1.39	1.39	1.94
		(0.00%)	(20.96%)	(30.70%)	(30.67%)	(39.18%)
404.6	Oxidized species	—	1.17	—	0.25	0.21
		(0.00%)	(28.76%)	(0.00%)	(5.53%)	(4.30%)
**N1s (at.%)**		26.62	4.06	4.52	4.52	4.96

**Table 3:** Results of the fit of the XPS O 1s region; values given in at.% (percentage total intensity).

Binding Energy (eV)	Functional Group	ZIF-8	ZDC-850	PAN	ZIF-8/PAN	ZDC-850/PAN
531.5	C=O/O=C-N	3.21	3.13	5.51	3.47	5.05
		(100.00%)	(71.66%)	(61.94%)	(54.00%)	(67.32%)
532.7	C-OH	—	—	1.00	1.49	0.79
		(0.00%)	(0.01%)	(11.19%)	(23.27%)	(10.48%)
533.7	COOH	—	1.24	2.39	1.46	1.66
		(0.00%)	(28.33%)	(26.88%)	(22.73%)	(22.19%)
**O1s (at.%)**		3.21	4.37	8.90	6.42	7.50

**Table 4:** Results of the fit of the XPS C 1s region; values given in at% (percentage of total intensity).

Binding Energy (eV)	Functional Group	ZIF-8	ZDC-850	PAN	ZIF-8/PAN	ZDC-850/PAN
285	C-C/C=C	44.96	42.57	47.67	39.66	61.35
		(79.92%)	(46.92%)	(55.06%)	(45.18%)	(70.07%)
286.1	C-N	10.20	22.86	13.22	38.04	1.00
		(18.13%)	(25.19%)	(15.27%)	(43.33%)	(1.14%)
286.6	C=N/C-OH	0.03	7.06	17.53	1.05	8.14
		(0.06%)	(7.78%)	(20.24%)	(1.19%)	(9.29%)
287.6	C=O	1.06	2.76	0.39	0.01	9.08
		(1.89%)	(3.04%)	(0.46%)	(0.01%)	(10.37%)
290.5	-COOH	—	15.49	7.77	9.03	7.99
		(0.00%)	(17.07%)	(8.97%)	(10.29%)	(9.12%)
**C1s (at.%)**		56.25	90.73	86.58	87.78	87.55

**Table 5:** Fitted results of the Freundlich equation for the CO_2_ adsorption data collected on the samples at different temperatures.

Sample	Temperature (°C)	K_F_ (mmol/g/kPa^1/n^)	*n*	R^2^
ZIF-8	25	0.0035	0.84	0.99997
	40	0.0020	0.80	0.99992
	55	0.0006	0.69	0.99723
ZDC-850	25	0.1389	1.43	0.99998
	40	0.1180	1.44	0.99995
	55	0.0577	1.30	0.99972
PAN	25	0.1782	1.65	0.99952
	40	0.0886	1.43	0.99960
	55	0.0567	1.32	0.99991
ZIF-8/PAN	25	0.1512	1.39	0.99865
	40	0.0546	1.18	0.99785
	55	0.0469	1.18	0.99661
ZDC-850/PAN	25	0.1955	1.62	0.99990
	40	0.1174	1.49	0.99995
	55	0.0667	1.35	0.99985

**Table 6:** Comparison of the CO_2_ uptakes of the adsorbents in this study with various ZIF-8-related adsorbents in the literature.

Adsorbent	S_BET_ (m^2^/g)	Conc. of CO_2_	Temp. (°C)	CO_2_ Uptake (mmol/g)	Reference
ZIF-8	1579	1 atm	25	0.88	This study
ZDC-850	1873	1 atm	25	3.50	This study
ZIF-8/PAN	1178	1 atm	25	4.20	This study
ZDC-850/PAN	1046	1 atm	25	3.38	This study
ZIF-8	1016	1 bar	25	0.60	[41]
ZIF-PAN	888	1 bar	25	0.28	[41]
ZIF-7-8s	283	1 bar	20	1.44	[45]
ZIF-8	1169	1 bar	0	1.41	[44]
			25	1.06	
NH_2_-ZIF-8	886	1 bar	0	2.12	[44]
			25	1.94	
NH_2_-ZIF-8/bacterial cellulose foams	455	1 bar	0	1.71	[44]
			25	1.63	
ZIF-100	595	1 atm	0	1.3	[8]
			25	0.6	

## Data Availability

The data presented in this study are available on request from the corresponding author. The data are not publicly available due to privacy restrictions.
